# Effectiveness of an osteopathic treatment on the autonomic nervous system: a systematic review of the literature

**DOI:** 10.1186/s40001-019-0394-5

**Published:** 2019-10-25

**Authors:** Verena Rechberger, Michael Biberschick, Jan Porthun

**Affiliations:** 1 Vienna School of Osteopathy, Donau University Krems, Vienna, Austria; 2 Therapiegemeinschaft Holochergasse, Vienna, Austria; 3 Department of Health Sciences Gjøvik, Norwegian University of Science and Technology, Gjøvik, Norway

**Keywords:** Osteopathy, Autonomic nervous system, Treatment techniques

## Abstract

**Objective:**

The objective of this systematic review was to evaluate the effectiveness of an osteopathic treatment on the autonomic nervous system (ANS). For this purpose, published primary studies were analysed and critically evaluated.

**Method:**

To generate this review, 15 electronic databases were systematically searched for studies. Randomized clinical controlled trials (RCT) and clinical controlled trials (CCT) are included in the review and evaluated with appropriate assessment tools (Downs and Black Checklist and the checklist from Kienle and Kiene).

**Results:**

23 published studies (10 RCT, 1 clinic multi-centre study, 1 CCT, 5 randomized cross-over studies, 5 randomized pilot studies and 1 single case study) are included in this review. The studies were evaluated with the assessment tools according to their quality. 3 studies are graded as high quality, 11 as moderate and 8 as low-quality studies.

**Conclusion:**

The included published studies represent a good level of evidence. Due to a small number of subjects and no follow-ups, the methodological quality is rated as moderate. A significant change on the ANS was shown in studies including High-Velocity Low-Amplitude Techniques (HVLAT). No statement could be drawn in studies in which they used cranial osteopathic techniques due to the lack of methodological quality. A significant change on the ANS is shown in the treatment of the suboccipital region. In studies which evaluated the effectiveness of mobilization in the cervical and thoracic region, no statement could be displayed due to a low level of evidence. None of the findings in these studies have given statements if ANS activation takes place in the sympathetic or parasympathetic system.

## Background

The osteopathic health care with its philosophy and principles of practice is a holistic, person-centred treatment concept with body structure (anatomy) and body function (physiology) as a unit [1].

The osteopathic treatment principles are integrated in the five osteopathic treatment models which are categorized as followed: postural—biomechanical model, respiratory—circulatory model, bioenergetic—metabolic model, biopsychosocial model and neurologic–anatomic model [2].

The treatment of the autonomic nervous system (ANS) is an important sequence of the treatment procedures as it is involved in the regulation of the homoeostasis of the body functions, e.g. cardiovascular system, blood pressure, temperature [3]. Therefore, a good function of the innervations from the 1st centre of the ANS to the target organ is required [3]. Imbalances between the sympathetic and parasympathetic over a longer period of time can lead to various symptoms in the body. It has an influence on, e.g. the vasoconstriction of the blood vessels in the gastro-intestinal region, vasodilatation in the skeletal muscles, low or high heart rate, changes in the secretion of glands, changes in the bronchial muscles and glands which can be revealed by the body as a malfunction of an organ [4].

A wide variety of manual treatment techniques is used by osteopaths to improve the physiological functions in the body and to regain homoeostasis which has been altered by somatic dysfunction [5]. Osteopathic manipulation techniques are defined as followed: thrust manipulation techniques (High-Velocity Low-Amplitude Thrust), soft tissue techniques (myofascial release techniques), Counterstrain techniques, Balanced Ligamentous Tension and Ligamentous Articular Strain techniques, visceral techniques, osteopathic craniosacral techniques [1].

Welch and Boone [6] reported in their study in 2008 that cervical and thoracic manipulation techniques influence the activity of the ANS. A rib raise technique, which Henderson et al. [7] discovered in their study, also has an influence on the ANS activity. Other osteopathic treatment techniques such as craniosacral compression techniques on the fourth ventricle of the brain also show a change in the ANS activity which Curi et al. [8] studied in 2017.

Unfortunately, the number of studies done in this field displays indifferent results in the change of the ANS activity.

The aim of the current review is to evaluate with the existing literature, if an osteopathic treatment affects the autonomic nervous system and if a certain treatment in different body regions has an effect on the sympathetic or parasympathetic nervous system and can lead to changes in different malfunctions in the body system as mentioned above. Study search was restricted by language barriers (only English, German) due to the writer’s language limitations.

## Main text

### Methods and materials

A comprehensive search of the existing literature was undertaken to identify published original research examining the effectiveness of treatments to the ANS. In accordance with the PRISMA statement, the review techniques in this study followed their standards [9]. The included studies for this review were carried out from researchers all over the world, e.g. Australia, Brazil, Canada, France, Italy, Japan, Malaysia, New Zealand, Spain, UK, USA.

#### Inclusion criteria

There were no time limits set for the research of the literature.

##### Types of studies

The present systematic review included randomized clinical controlled trials (RCT), clinical controlled trials (CCT), single case studies, multi-centre studies, pilot studies and observational studies.

##### Types of interventions

All studies in the field of osteopathic manipulative treatments (OMT), physiotherapy, chiropractic, manual medicine were included in the search strategy. Some treatment techniques in these professional groups overlap, which is why they were all included in the search.

#### Exclusion criteria

Articles were excluded if they were not written in English or German, did not present original empirical data or only an abstract was available or if they only represented active physiotherapy in the study. Also excluded were articles in which OMT was combined with other therapy forms. Conference proceedings, editorials, letters or seminar dossiers were excluded too.

#### Outcomes

The primary outcome was to estimate the efficacy or effectiveness of an osteopathic treatment on the ANS. As a second outcome, the effect of treatment techniques in different body regions for sympathetic and parasympathetic nervous system was evaluated. Any kind of an adverse event was a second outcome.

#### Data sources and searches

A systematic literature search was performed from March 12, 2018 to April 14, 2018 in the following electronic databases: PubMed, Chiroindex, The Cochrane Library, BioMedCentral, Osteomed Dr., EBSCO-psycharticles, PEDRO, Springerlink, Embase-Elselvier, LIVIVO, ScienceDirect, AMED, EBSCO-CHINHAL, pubpsych.zipd.de, Chiropractic & Manual Therapies.

The following search terms, defined by MeSH (Medical subject headings), were used: osteopathic medicine (OM), autonomic nervous system (ANS), parasympathetic nervous system (PNS), sympathetic nervous system (SNS), musculoskeletal manipulations, chiropractic, therapy/therapeutic use, manual therapy, mobilization. The search was supplemented by a manual search in the reference list for all relevant studies that were not listed when searching the electronic databases and by citation tracking of the identified trials.

##### Study selection

The author of this review systematically screened the titles and abstracts of the results from the search strategy (1st selection). Potential studies were read in full text and once more evaluated for the inclusion in the current review (2nd selection).

##### Data extraction

Data were extracted by the author of the current review. For the purpose of this review, the information extracted from the included papers were author, source, and design, characteristics of the participants/aim of the study, sample, intervention, and summary of findings.

##### Dealing with missing data

If the article did not contain sufficient information or was not completed, the authors were contacted for additional information or the full article.

##### Quality assessment

The summary of results was reported following the PRISMA statement [9].

The quality of evidence for each outcome in the included studies was assessed using the Downs and Black scale [10] for RCT, non-RCT studies, and for the single case studies, the checklist from Kienle and Kiene was used [11].

The Downs and Black scale is based on a checklist of 27 items and has been found to be valid for critically evaluating RCT and non-RCT studies [10]. The checklist included four categories for evaluation: reporting, external validity, internal validity/bias, internal validity/confounding. The Downs and Black scale specifies 4 levels of quality using this scoring system which is categorized as followed in Table 1: strong, moderate, limited or poor quality [12].

**Table 1:** Categorization of total scores of the Downs and Black Checklist [12]

Quality index	Percentage	Methodological quality score (n = 27)
Strong	≥ 75%	≥ 21
Moderate	50–74%	14–20
Limited	25–49%	7–13
Poor	< 25%	< 7

The checklist from Kienle and Kiene was developed to evaluate the methodological quality of single case studies, case series [11]. It consists of 11 items which are scored with zero or one point. The quality index of the scoring system is categorized with good, moderate or poor as shown in Table 2.

**Table 2:** Categorization of total scores obtained by Kienle and Kiene [11]

Total score	Quality index
11–9 points	Good
9–5 points	Moderate
5–1 points	Poor

### Results

#### Included studies

**Fig. 1:**
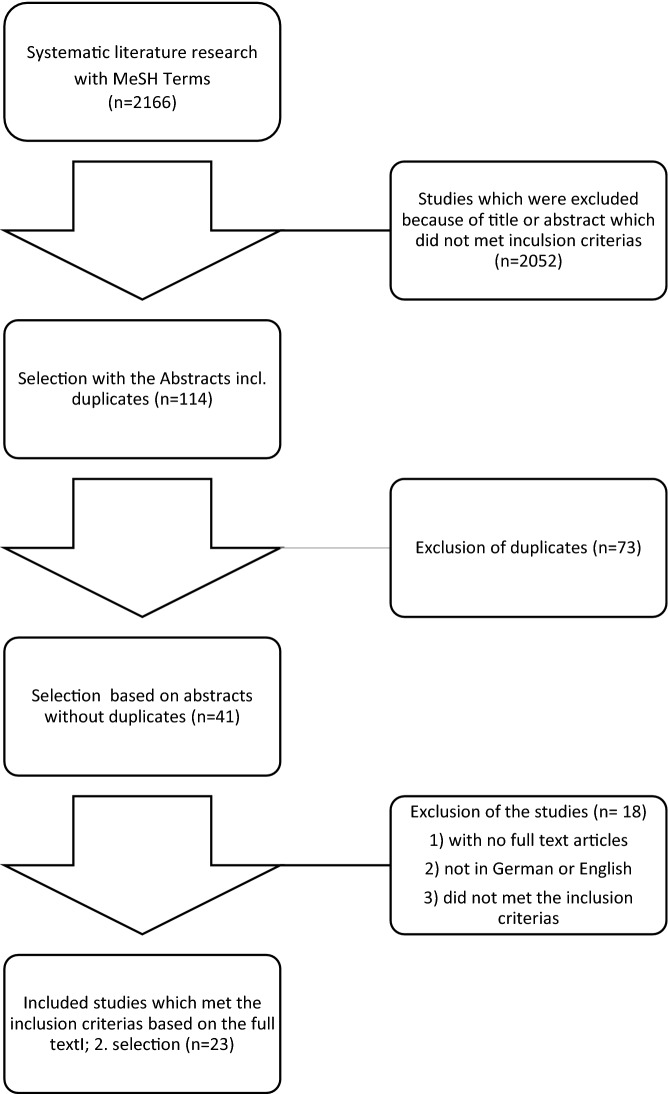
Flow chart of the study selection

Electronic search identified 2166 papers (see Fig. 1). In the first selection process after screening title and abstracts, 114 studies were left (1st selection, including duplicates). In the next step, 73 duplicates were removed. After reading the full text of 41 papers and applying the predefined inclusion criteria, 23 articles were left for the qualitative analysing (2nd selection).

#### Excluded studies

In the first selection process, 73 duplicates were excluded and from the 41 papers read in full text, 18 were excluded for various reasons.

##### Study details

Of the included articles, one study [13] performed an OMT intervention based on each patient’s needs and 6 studies [8, 14–18] dealing with cranial osteopathic manipulative medicine (OMM). 15 of the 23 studies were dealing with a healthy population. In the other articles, the included subjects were dealing with craniofascial pain and temporomandibular dysfunction (TMD) [19], hypertensive and normotensive blood pressure [8], lumbar pain vs. pain free [20], acute cervical pain vs. pain free [21], lumbar pain, cervical pain or headache [22], acute back pain [23], cervical pain and stiffness [24] and certain symptomatic region [25].

Globally, a wide range of techniques was used. The following treatment techniques were investigated: mobilization of the cervical spine [19–21], posterior/anterior mobilization of the thoracic spine [28], mobilization of the lumbar spine [23], soft tissue techniques in the cervical spine region [24, 25], HVLAT techniques in the thoracic spine [6, 15, 26], HVLAT techniques in the lumbar spine [23, 26–28], HVLAT techniques in the cervical spine [6, 26, 28–32] and a rib raise technique [7].

The results of the quality of the included studies scaled with the Downs and Black checklist are listed in Table 3. The single case study [25], evaluated with the checklist from Kienle and Kiene [11], scored 7 points and the result on the quality index was moderate.

**Table 3:** Rating of the studies with the Downs and Black Scale [10]

Authors	Study types	Score	Quality index
Budgell and Hirano [31]	Rand. cross-over design	16	Moderate
Cardoso-de-Mello-e-Mello-Ribeiro [18]	RCT	18	Moderate
Chiu and Wright [27]	RCT	11	Limited
Curi et al. [8]	RCT	16	Moderate
Fornari [14]	Rand. pilot study	18	Moderate
Fukada et al. [24]	Rand. cross-over design	13	Limited
Gibbons et al. [32]	Rand. pilot study	13	Limited
Giles et al. [29]	Rand. cross-over design	13	Limited
Henderson et al. [7]	Rand. pilot study	17	Moderate
Jowsey and Perry [28]	RCT	18	Moderate
La Touche et al. [19]	RCT	24	Strong
Milnes and Moran [16]	Pilot study	12	Limited
Petersen et al. [26]	RCT	13	Limited
Purdy et al. [30]	Rand. cross-over design	14	Moderate
Roy et al. [20]	RCT	19	Moderate
Ruffini et al. [13]	RCT	23	Strong
Shi et al. [17]	Rand. cross-over design	16	Moderate
Scoppa et al. [15]	Rand. pilot study	10	Limited
Win et al. [21]	RCT	21	Strong
Welch and Boone [6]	CCT	10	Limited
Younes et al. [23]	RCT	20	Moderate
Zhang et al. [27]	Clin. multi-centre study	15	Moderate

The studied population ranged in size from 1 in the single case study [25] and 10 in a pilot study [16] to 539 in a clinical multi-centre study [22]. Treatment duration ranged from 3 times 1 min [26] to 45 min [23]. The length of treatment period differed, ranging from single treatments to regularly treatments in a period of 5 weeks. The time when measurements were taken to evaluate changes in the ANS after the treatment differs in all studies. They were taken immediately after the treatment or have follow-up measurements a few times after in different intervals (280 min after intervention).

A wide range of measurements can be used to demonstrate changes in the ANS through heart rate variability (HRV) [14, 15, 23] (quantitative marker for changes in the vegetative nervous system [33]), heart frequency, blood pressure [23], breathing rate [17], edge light pupil effect [32], distal skin conductance/skin temperature [20, 21, 25], and also through changes in the immune [24] and endocrine system [6, 7].

The participants in the study groups were healthy adults or patients with pain in the body region that was tested in the study.

The subject groups in the included articles for this review were very heterogeneous and therefore, no statistical analysis of the study results was possible in this review.

##### Effects of interventions

Table 4 provides a summary of the characteristics of each study, including treatment sessions and interventions.

**Table 4:** Main characteristics of included studies

Author	Source	Study design	Aim of the study/characteristics of the study participants	*n* (?)	Intervention	Results
Ruffini et al. [13]	Frontiers in Neuroscience	RCT	Effect of an OMT in healthy subjects compared to control group and sham therapy measured with variations of high-frequency (HF) parameter of HRV (2 × a week);	*n* = 66	Two therapies for 25 min (10 min evaluation; 15 min patient need-based OMT) Group A: OMT + sham therapy Group B: sham therapy + OMT Group C: 2× no intervention	Statistic sign. difference in the groups in HF (primary outcome measure p < 0.001) and HRV outcome measures. No difference in sham therapy and in Group C with no intervention
Fornari [14]	JAOA	Randomized pilot study	To explore the modulating effect of cranial OMT on healthy young men on autonomic neural regulation measured with HRV and to verify its ability to influence the activity of the hypothalamic–pituitary–adrenocortical axis (measured with saliva samples)	*n* = 20	Immediate cranial OMT after an acute 5-min mental stress episode for the intervention group; control group received sham therapy with light touch	Statistic sign. reduction of chronotrop effect with OMT after an acute stressor (t = − 2.9, p < 0.05) and modulation of sympathovagal balance towards sympathetic activity (t = − 2.8, p < 0.05); lower cortisol level after acute mental stressor in intervention group (t = − 2.3, p < 0.05)
La Touche et al. [19]	The Clinical Journal of Pain	RCT	The aims were to investigate the effects of an anterior-posterior upper cervical mobilization on pain modulation in craniofacial and cervical regions and its influence on the sympathetic nervous system	*n* = 32	Anterior–posterior mobilization (C0–C3) with 0.5 Hz, 3 intervals with 2 min each, 30-s pause in between; Control group received sham therapy	Increased pressure pain thresholds in the craniofacial and cervical regions in intervention group (p < 0.001), and decrease in pain intensity in the intervention group compared with placebo (p < 0.001). A change in the sympathetic nervous system is due to pain relief
Petersen et al. [26]	Physiotherapy Theory and Practice	RCT	The aim of this study was to evaluate the effect of a commonly utilised cervical mobilization technique on sympathetic function in the upper limb of normal pain-free volunteers	*n* = 16	Each participant was in the intervention, control and placebo group. Duration: 3 times 1 min on the vertebra C5 (grade III)	The mobilization (grade III) with healthy subjects stimulates the periphery sympathetic nervous system and increases the skin conductance (50–60%) (p < 0.05); no statistical change in skin temperature (p = 0.10)
Jowsey and Perry [28]	Manual Therapy	RCT	This study investigated whether a grade III posterior-anterior rotator joint mobilization technique applied on T4 vertebra at a frequency of 0.5 Hz had demonstrably greater effects than placebo intervention on skin conductance in the hands of healthy subjects	*n* = 36	Intervertebral grade III p/a rotator mobilization 3 × 1 min	A grade III p/a rotator mobilization technique shows a side-specific increase in the sympathetic activity in the right hand (F = 4.888, df = 35, p = 0.034).
Giles et al. [29]	The Journal of Alternative and Complementary Medicine	Randomized cross-over design	The study was designed to determine the acute effect of upper cervical spine manipulation (soft tissue techniques and sub occipital decompression) in healthy subjects on the ANS measured by HRV	*n* = 19	Each participant acts as its own intervention, control and placebo group; Duration of therapy: 15 min	The results show a modulation in the parasympathetic HRV measurements and a shift to sympathovagal balance. Increase in the SDNN interval (p < 0.01); increase in the HF in spectral power (p = 0.03) Decrease in LF/HF ratio (p = 0.01)
Scoppa et al. [15]	Acta Medica Mediterranea	Randomized pilot study	The purpose of this pilot study with healthy subjects was twofold: Compare different OMTs that supposedly interact either with ortho- or para-sympathetic branches of the ANS Ascertain whether there is a relationship between autonomic Balance and Quiet Upright Stance Balance	*n* = 51 randomized to different groups	HVLAT on the dorsal spine or CV4 technique (10 min); Time measurement: immediate after OMT, after 20 min, after 100 min, after 280 min; balance test with each measurement	Immediate reduction in ANS after HVLAT. After CV technique delayed response in the ANS. Both treatments have an influence on the quiet upright stance balance
Purdy et al. [30]	JAOA	Randomized Cross-over design	Evaluation of the effect of a soft tissue technique in the suboccipital region measured by blood flow in the finger as a marker for the sympathetic nervous system change	*n* = 25	Each subject was in intervention (A) and sham (B) group. 120-s treatment either A or B; 10-min Pause, then the other treatment	All groups had sign. changes in the measurement with OMT. As a conclusion, there is a sign. reduction in the sympathetic nervous system activity in group A (change in X: p = 0.01, Y: p < 0.001) and B (change in X: p = 0.0014, Y: p = < 0.0001). No change was with subjects who felt uncomfortable with the treatment
Milnes and Moran [16]	International Journal of Osteopathic Medicine	Pilot study	The aim of this study was to investigate the physiological effects in healthy subjects resulting from the administration of a single cranial technique (CV4) compared with simple touch	*n* = 10	Design with repeated measurements; Each subject acts in intervention and placebo group. 5-min touch only (placebo), CV4 treatment according to patients’ need	No statistic difference is shown in skin temperature and HRV with ANOVA Constant breath frequency in all 5 phases MW: 14.12 (SD 3.2) Concluding to the results, there is only a minimal physiological effect with a CV4 technique on the ANS. There might be “responder” and “non-responder” for cranial OMT.
Shi et al. [17]	JAOA	randomized Cross-over design	The aim of this study is to examine the effects of cranial OMT on cerebral tissue oxygen saturation (SctO2) and the ANS in healthy adults	*n* = 21	Each subject acts in intervention and placebo group. 2 cranial OMT techniques (augmentation and suppression) each for 4 min	Statistical sign. reduction of the SctO2 during OMT (left: p = 0.026, right: p = 0.007) suppression technique and through this a balance in the ANS. No effects with augmentation or placebo. Decrease in LF and increase in HF (p = 0.05)
Cardoso-de-Mello-e-Mello-Ribeiro [18]	Evidence-Based Complementary and Alternative Medicine	RCT	This study aimed at evaluating the effects of a CV4 compression technique on the ANS	*n* = 40	Intervention group received CV4 technique for 10 min; placebo group: bilateral non-therapeutic contact on the occipital bone for 10 min	CV4 compression technique seems not to have any effect in plasmatic catecholamine levels, blood pressure or heart rate. No sign. change in the comparison of pre- or post-intervention results in all study groups (p < 0.01)
Curi et al. [8]	Journal of Bodywork and Movement Therapies	RCT	The aim of this study was to compare blood pressure and HRV amongst hypertensive stage I (HT) and normotensive (NT) individuals who were submitted to cranial CV4 technique	*n* = 30	CV4 technique was applied to both groups. Measurements were taken immediate after technique, 5, 10 and 15 min after intervention	Blood pressure reduction in HT group, statistical sign. changes in sympathovagal tension after 15 min (measured with SDNN: p = 0.01) in both groups. RMSSD measure changes in the HT group pre- and post-intervention (p = 0.01) The data showed a change in the sympathovagal balance.
Chiu and Wright [27]	Manual Therapy	RCT	The aim of this study was to compare the effects of different rates of application of two different rates (2 Hz or 0.5 Hz) of a C5 grade III central posterior–anterior mobilization technique on skin conductance and skin temperature on sympathetic function in normal pain-free volunteers	*n* = 16	Treatment applied on 3 successive days. Randomized intervention group 1 (2 Hz) or intervention group 2 (0.5 Hz) or placebo each for 1 min	There is a sign. change in the SC between group 1 and 2 and a sign. effect in the ST in all 3 groups. The mobilization grade III with 2 Hz shows an increase in SC (50–60%) and an increase in efferent sympathetic activity.
Roy et al. [20]	Journal of Manipulative and Physiological Therapeutics	RCT	The purpose of this study was to examine the HRV in the presence of absence of pain in the lower back, whilst receiving one chiropractic treatment at L5 from either a manually assisted mechanical force (Activator) or a traditional HVLAT	*n* = 51	Intervention group 1: thrust with Activator, sham group 1: contact only with Activator, Intervention group 2: HVLAT on L5 (subject with pain), sham group 2: only lumbar roll position, no thrust; Control group: no intervention	In all groups except in the control group, the HRV was decreased. There is a change in the parasympathetic activity after a HVLAT or Activator technique in the lumbar region. Group differences in HRV modulation depend on subjects with or without pain
Win et al. [21]	Journal of Chiropractic Medicine	RCT	The aims of this study were to examine ANS response by HRV hemodynamic parameters and numeric pain scale when either upper (C1 and C2) or lower (C6 and C7) cervical segments were manipulated in healthy subjects or in patients with acute neck pain	*n* = 30	Week 1: data collection; Week 2: randomized to upper or lower cervical intervention; Week 3: other missing cervical intervention	According to the statistical changes in HRV parameters, there is a change in the parasympathetic system after intervention of the upper cervical spine (increase in SDNN: p = 0.02) and a change in the sympathetic system after invention in the lower cervical spine (decrease in systolic BP p = 0.002). A dominant parasympathetic activity is shown in patients with acute neck pain in the upper and lower cervical spine.
Zhang et al. [27]	Journal of Manipulative and Physiological Therapeutics	Clinical multi-centre study	The purpose of this study is to investigate the effect of a 4-week chiropractic care in a multiclinic setting on ANS activities using HRV with patients suffering from lumbar or neck pain or headache	Data collection from 96 therapists; *n* = 539	Intervention: in 4 weeks, 1 chiropractic treatment once a week; placebo: no intervention	Statistic sign. change in HRV (SDNN, VLF, total power, LF: P < 0.05) after 1 chiropractic treatment. After 4 weeks, there is also a change in HRV due to a change in ANS activity.
Budgell and Hirano [31]	Autonomic Neuroscience	Randomized Cross-over design	Effects of a cervical HVLAT technique (C1–C2) on HRV in healthy subjects with a 1-week pause in between intervention and placebo	*n* = 25	Randomized allocation in which week intervention or placebo takes place	Intervention group showed a sign. change in HRV parameters (absolute LF: p = 0.916, normal LF: p = 0.0061, LF/HF ratio: p = 0.0037) which may reflect a shift in balance between sympathetic and parasympathetic output to the heart.
Younes et al. [23]	Chiropractic & Manual Therapies	RCT	The study aimed to quantify the effect of an OMT at patients with acute back pain on the parasympathetic autonomic control measured with HRV, baroreflex, systolic blood pressure	*n* = 22	45-min intervention: HVLAT, lumbar mobilization, deep massage, Strain–Counterstrain techniques, MET, ischaemic pressure massage. 2 times (1st and 7th day)	Sign. increase in IG: RMSSD (p = 0.003), HF (p = 0.005) Baroreflex sensitivity in HF-band higher in IG than in CG (p < 0.001) A vagal modulation of the HRV in the intervention group took place.
Welch and Boone [6]	Journal of Chiropractic Medicine	CCT	The aim of this study was to measure the effect of a cervical or thoracic HVLAT technique in healthy subjects on the ANS measured with blood pressure, pulse rate and HRV	*n* = 40	2 times HVLAT intervention (cervical and thoracic spine)	A sign. parasympathetic activity after cervical HVLAT (decrease of diastolic BP: P = 0.038, moderate clinical effect (0.50), increase in pulse rate: P = 0.044) and a sign. sympathetic activity after thoracic HVALT intervention. Unfortunately, HRV was measured only with few patients.
Fukada et al. [24]	Alternative Therapies	Cross-over design	Investigation of cerebral metabolic changes after a chiropractic spinal manipulation in subjects with cervical pain and neck stiffness measured with FDG-PET	*n* = 15	Intervention group: cervical manipulation with Activator and PET scan, placebo: group PET scan only.	There is a sympathetic relaxation and pain reduction after spinal manipulation because of a change in the cerebral glucose metabolism (P < 0.001)
Henderson et al. [7]	JAOA	Randomized pilot study	Effects of a rib raise technique on the ANS and the hypothalamic–pituitary–adrenal axis with healthy subjects measured with salivary flow rate, Alpha amylase activity and cortisol level	*n* = 23	Intervention or sham therapy	Rib raise technique decreases the sympathetic activity (immediate reduction in salivary alpha amylase activity (p = 0.014) and 10 min after (p = 0.008)), but there is no influence on the hypothalamic–pituitary–adrenal axis because there is no change in parasympathetic activity.
Gibbons et al. [32]	Journal of Manipulative and Physiological Therapeutics	Randomized pilot study	The aim of this study was to investigate a HVLAT technique in the cervical spine (C1 + C2) and their effects on the ANS measured by ELPCT (edge light pupil cycle time) in healthy subjects	*n* = 13	Randomized assignment if HVLAT rotator component was to the right or left. No control group	Sign. change in ELPCT before and after HVLAT in both eyes (p = 0.002) There is a change in the ANS after HVLAT technique in the cervical spine because the ELPCT is mediated through the ANS.
Driscoll and Hall [25]	Journal of Manipulative and Physiological Therapeutics	Single case study	The aim of this study was to evaluate if there is a change in the ANS and cardiovascular system after chiropractic HVLAT technique in the symptomatic regions of the spine measured with blood pressure, pulse rate and ECG	*n* = 1	10 treatments in 5 weeks	There was a change in parasympathetic and sympathetic HRV measures in different treatments. No sign. change in the measurements of the diastolic and systolic blood pressure.

### Discussion

The general quality of the selected articles was good including a lot of RCTs. This is why the author concluded that overall there is a positive result towards the question if an osteopathic treatment can influence the ANS.

As the treatment techniques and the treated body regions in the included studies vary a lot, the included studies were summarized in groups for the discussion. Therefore, the heterogeneity of the articles does not allow for a statistical analysis.

### High-velocity low-amplitude techniques (HVLAT) in the cervical spine

Six included articles [6, 21, 22, 24, 31, 32] investigated the effects of HVLAT techniques in the cervical spine on the ANS. Techniques in the upper and lower cervical spine in one article [21], which is categorized as strong in the Downs and Black checklist [10] and including 30 subjects show statistical changes in the ANS. Findings in another article [31] are that techniques in the upper cervical region on 25 healthy subjects expose significant changes in the ANS. In a randomized cross-over design study [24], which was assessed in limited methodological quality, changes were measured in the ANS through sympathetic relaxation. Inconsistent results are shown in one article [6], because their measurement tool varies (HRV, blood pressure, pulse rate). Only 7 out of 40 were measured with HRV, which qualifies as the gold standard. Their results show an increased parasympathetic activity in cervical techniques and sympathetic in thoracic techniques. In the randomized pilot study [32], changes in the ANS through techniques in the upper cervical region on 13 subjects were measured by use of the edge light pupil cycle time. This study points at a low level of evidence and was categorized as limited in their methodological quality. In a multisite clinical study [22], 539 subjects with cervical and lumbar pain and headache were measured with HRV after a HVLAT technique in the pain region. Despite a moderate categorization in the methodological quality, there are significant changes in the ANS measured.

Although the studies were limited regarding number of subjects, methodological quality and level of evidence, there is a significant change in the ANS in all publications. Almost all used HRV as a measurement instrument. No valid statement is possible if sympathetic or parasympathetic is more influenced by HVLAT techniques in the upper or lower cervical spine.

#### HVLAT in the lumbar region

2 studies included [20, 23] investigated the effects of HVLAT techniques in the lumbar spine. In one study [23], the effect with 22 subjects with acute low back pain was measured through a significant change in the modulation of the ANS. In another article [20] with a good level of evidence and a moderate categorization in the methodological quality, the effect of a HVLAT technique on L5 shows a significant change in the parasympathetic activity.

In addition in a single case study [25], there were also changes in the ANS after using a HVLAT technique on the lumbar spine.

#### Cranial OMT techniques

In 8 studies [8, 14–18, 29, 30], the influence of a cranial OMT technique on the ANS was examined.

In an article [8], they measured changes by means of HRV and blood pressure in 30 subjects, half of them were normotensive and the other half hypertensive, with a cranial technique named CV4. The results showed a significant change in the parasympathetic activity. No follow-up was taken and no conclusion for a parasympathetic long-term effect with hypertension is possible. In one RCT [18], they compared the effect of a cranial CV4 technique and a placebo in 40 subjects. Measurement is taken with plasmatic catecholamine levels, blood pressure and heart rate. No effects related to the intervention were found. Another study [17] measured the effect of a cranial osteopathic technique by the use of HRV and cerebral tissue oxygen saturation in healthy subjects. The results show an effect in both measurement tools and therefore changes in the ANS. In an article [30] with 25 subjects, they measured the blood flow in the finger after a gentle soft tissue manipulation in the suboccipital region. Results show a slight reduction of the sympathetic activity. The level of evidence is low and the methodological quality is moderate, so the results have to be interpreted with care. In a randomized cross-over design [29], it was studied if a so-called suboccipital decompression and a soft tissue technique in this region on 19 subjects influence the parasympathethic system and the N. Vagus measured by HRV. Results show a significant change in the ANS. The methodological quality is limited and the number of participants is small which lowers the evaluation. In a pilot study [14], 20 subjects were treated with craniosacral techniques after an acute stressor. Changes were measured by HRV and cortisol level samples in saliva. The results show a reduction on the ANS and a prevention of the increase in the cortisol level. A pilot study [16] compared the effects of a craniosacral CV4 technique and a placebo therapy. The results on 10 subjects showed a very low impact on the ANS. In a pilot study [15] it was measured, if a HVLAT in the thoracic spine region or a craniosacrale CV4 technique shows more changes in the ANS. The results show that a CV4 technique influences sympathovagal balance and the HVLAT reduces the sympathetic system.

According to all the results in these studies, there is a tendency to a positive change in the ANS. A significant change was not reported. Only techniques in the suboccipital region showed significant changes in the ANS.

#### Mobilization techniques in the thoracic and cervical spine

In 4 studies [19, 26–28], the influence of mobilization techniques in thoracic or cervical spine areas on the ANS measured through changes in the upper extremity was examined. Results of this investigations show that there is an alteration in the vegetative nervous system. No valid statement is possible if it is in the 1st, 2nd, or 3rd centre of the ANS. An RCT study [19] measured the effect of an anterior/posterior mobilization of the upper cervical spine on the pain reduction in the craniofascial region and on the TMD. The pain reduction is related to changes in the sympathetic nervous system. The study has a good level of evidence. In another RCT [28], they investigated the effect of a posterior/anterior rotation mobilization in the thoracic spine. Significant changes in the sympathetic nervous system were measured in the hands. The level of evidence of this study is good and the methodological quality was rated moderate. In one article [26], they measured the effect of mobilization of the vertebra C5 on the sympathetic function of the upper extremity. The results show a significant increase in the skin conductance. The results are limited as the methodological quality is poor and the number of probands is only 16. The effect of posterior/anterior mobilization in the cervical spine with 0.5 Hz or 2 Hz on the sympathetic function of the upper extremity was investigated in an RCT [27]. The result shows a change in the skin conductance which means an increase in the efferent sympathetic activity. Considering the outcomes of the study, it has to be said that the results of the measurement parameters are very inconsistent.

Two [7, 13] additional studies, which were not summarized in one of the groups above, are discussed in the following text paragraph.

In an RCT [13], they investigated the effect of an osteopathic treatment on 66 subjects with placebo and control group measured by HRV. Results show a statistical significant change in the ANS.

In a randomized pilot study [7], the effect of a rib raise technique on the ANS and the hypothalamic–pituitary–adrenal axis using non-invasive biomarkers was investigated. The results suggest that there is a change on the ANS, the methodological quality is moderate and the level of evidence is good.

### Conclusions

Results from this review showed that studies on the effectiveness of OMT on the ANS are scarce in subjects, heterogeneous and limited in the methodological quality. Nevertheless, a conclusion if the efficacy of OMT is given can be answered positively. No conclusive statement if the ANS can be influenced by cranial OMT can be reported. Also, no declaration can be made if a certain treatment in an area can have more influence on the sympathetic or parasympathetic nervous system.

Further, high-quality research with a larger number of subjects should be done in this field.
